# Development of a Clinic-Based, Sociostructural Intervention to Improve the Provision of Pre-Exposure Prophylaxis for Cisgender Women: Formative Study Using the Assessment, Decision, Adaptation, Production, Topical Experts, Integration, Training, and Testing (ADAPT-ITT) Framework

**DOI:** 10.2196/75922

**Published:** 2025-08-06

**Authors:** Rachel Katherine Scott, Shawnika J Hull, Deanna Kerrigan, Mandi Pratt-Chapman, Tara Mathias-Prabhu, Jennifer Zack, Michelle Xu, Marisa Sadauskas, Patricia L Moriarty, Tranessa L Hanson, Hannah Arem

**Affiliations:** 1 MedStar Health Washington, DC United States; 2 School of Medicine Georgetown University Washington, DC United States; 3 Rutgers, The State University of New Jersey New Brunswick, NJ United States; 4 George Washington University Washington, DC United States; 5 School of Medicine Saint Louis University St. Louis, MO United States

**Keywords:** pre-exposure prophylaxis, female patient, HIV infections, ambulatory care facilities, primary prevention, education

## Abstract

**Background:**

Cisgender women (subsequently referred to as women) account for 23% of new HIV diagnoses in the United States. There are significant sociostructural barriers to engagement and retention in the pre-exposure prophylaxis (PrEP) cascade, particularly for Black women.

**Objective:**

In response to the lack of evidence-based interventions (EBIs) to improve PrEP initiation, adherence, and persistence among women in the United States, we developed a clinic-based, sociostructural intervention focused on engagement and retention in the PrEP cascade for women.

**Methods:**

We used the Assessment, Decision, Adaptation, Production, Topical experts, Integration, Training, and Testing (ADAPT-ITT) model to adapt two Centers for Disease Control and Prevention (CDC) best practices in HIV prevention: HIV PrEP Services for Urban Women and Project Shikamana to create a culturally appropriate EBI responsive to Black women’s HIV prevention needs. In this paper, we focus on the first 6 steps of iterative adaptation in preparation for pilot testing. We conducted semistructured interviews and focus group discussions with key populations to inform and guide intervention development and used theater testing to evaluate the mock-up of the prototype. We conducted rapid qualitative analysis to identify key themes related to delivering PrEP to the intended population and engaged subject matter experts to refine the prototype.

**Results:**

For the *Assessment phase*, we conducted 10 in-depth interviews with key informants from community-based and HIV-prevention organizations and led 7 focus group discussions (n=4-8) to guide intervention development among health care providers (n=2 groups), PrEP navigators and educators (n=1 group), and female patients (n=4 groups). Key themes included population-specific barriers to PrEP use, namely accessibility and availability, perceived risk, and stigma. In addition, participants advised on model adaptation specific to PrEP navigation, clinic-level training, and social support. For the *Decision phase*, we selected 2 EBIs from the CDC HIV Compendium of Best Practices. For the *Adaptation phase*, we adapted and theater tested a preliminary intervention for feedback. For the *Production phase*, using feedback from theater testing, we created a prototype of the Women’s PrEP Project (W-PrEP) designed to address patient-, provider-, and clinic-level barriers to the provision of PrEP for women through a clinic-wide intervention delivering education, resources, and support (including PrEP navigation). For the *Topical experts* and *Integration phases*, we collected iterative feedback from our advisory board and subject matter experts and integrated feedback into the final prototype.

**Conclusions:**

The adaptation of the W-PrEP integrated key elements of the local context for women with potential exposure to HIV, as well as health care providers, clinic staff, and PrEP navigators. Next steps include training clinic staff in a real-world setting to pilot-test the acceptability, feasibility, and preliminary effectiveness of the intervention.

## Introduction

### Background: HIV Prevention for Women in the United States

Cisgender women (subsequently referred to as “women”) account for 23% of new HIV diagnoses in the United States [1], highlighting the unrealized opportunity for tailored HIV prevention programs in this population. The HIV epidemic in the United States disproportionately affects Black women of reproductive age [1-3]. Among women, HIV transmission occurs primarily through heterosexual transmission (84%), principally receptive vaginal sex [4,5]. HIV is preventable through consistent use of barrier contraception and HIV pre-exposure prophylaxis (PrEP) [6-12]. Barrier contraception, specifically male condom use, is notably limited by partner power discordance and is implicitly dependent upon partner cooperation [13].

Both daily oral and long-acting injectable formulations of PrEP offer women-controlled options for HIV prevention before risk exposure, circumventing the need for cooperation from sex partners. Pharmacoprevention with either daily oral tenofovir disoproxil fumarate and emtricitabine or injectable bimonthly cabotegravir decreases HIV transmission by up to 99% in women [7-11]. Despite the efficacy of PrEP, the number of prescriptions of PrEP to women in the United States remains dwarfed by the number of women with indications for PrEP [14], highlighting the considerable unmet need for biomedical HIV prevention among women, especially Black women [15-19].

There are significant sociostructural barriers to engagement and retention in the PrEP cascade for women, including medical mistrust, cost, stigma, and lack of access [20-28]. Our previous research predominantly among Black women of reproductive age with demonstrable potential exposure to HIV [29-32] highlights the critical importance of health care provider [20,23,25,26,28,33-38] and community or peer support [20,25,26,28,35-37] in engagement and retention in the PrEP cascade. The Centers for Disease Control and Prevention (CDC) further highlighted the importance of health care providers as PrEP educators with their 2021 guidance that providers should educate all sexually active adults and adolescents about PrEP [39]. Despite their pivotal role, health care provider knowledge of and comfort with prescribing PrEP continues to lag across medical specialties [22,40-45], and despite the synergy of offering integrated PrEP services as part of routine women’s health care, most women’s health clinics do not routinely educate about or prescribe PrEP [23,44-46]. Both our formative work and the existing literature indicate the sociostructural nature of the factors influencing women’s engagement in the PrEP cascade; thus, we used the socioecological model as our guiding theoretical framework for intervention adaptation.

### Theoretical Framework: the Socioecological Model

The socioecological model theorizes that an individual’s decisions and behaviors result from reciprocal interactions within and between the individual and their social, cultural, and structural environments [47]. Although health-related decisions or behaviors occur at the individual level, they are influenced by individual (ie, psychological), interpersonal (eg, relationship power and relationship commitment), community (ie, cultural norms and stigma), institutional (eg, equitable provision of care and appropriate services), and structural factors (eg, public policy and infrastructure) [48]. As applied to the PrEP cascade for women, our previously published survey of predominantly Black women found the intention to initiate PrEP was associated with different factors by level: at the individual level, positive attitudes toward PrEP and higher self-efficacy; at the community level, perceived acceptability of PrEP among peers and low fear of shame or stigma; and at the institutional level, having discussed PrEP with a health care provider [29]. Our findings underscore the importance of multilevel, clinic-based interventions for women, with education and tools to support women’s health providers as well as PrEP navigation to destigmatize PrEP and break down logistical barriers to PrEP initiation and continuation.

### Base Cases for Adaptation and Intervention Development

Given the critical role of providers as both PrEP educators and gatekeepers, we previously developed and tested HIV PrEP Services for Urban Women, a clinic-level educational intervention*,* which used a clinic-wide training and a provider toolkit to promote the provision of universal PrEP services. The intervention significantly improved the provision of PrEP services and PrEP uptake among women at a sexual health center in Washington, DC compared to baseline and was cited as an exemplar evidence-informed intervention (EII) in the CDC Compendium of Evidence-Based Interventions and Best Practices for HIV Prevention [49,50]. While the HIV PrEP Services for Urban Women EII significantly improved the provision of PrEP by providers and PrEP uptake among women of color of reproductive age, it did not actively address the social barriers of stigma, lack of community or peer support, or medical mistrust that the literature indicated were critical to engagement and retention in the PrEP cascade.

In contrast, Project Shikamana was a sociostructural intervention, also developed by a member of our research team, which showed decreased HIV acquisition among female sex workers in Tanzania and was designated by the CDC as an evidence-based intervention (EBI) for HIV prevention [50-52]. Project Shikamana’s strengths include its community-driven, multilevel approach to HIV prevention, involving provider sensitivity training, peer education, navigation and support, community mobilization to address sociostructural factors such as stigma and discrimination, and a focus on women of reproductive age. Given notable differences between the intended populations and the medical infrastructure and medical culture in Tanzania compared to the United States, we chose an evidence-based framework to adapt Project Shikamana while retaining its core strengths.

### This Formative Study

In response to the lack of EBI to improve PrEP initiation, adherence, and persistence among women in the United States, we adapted and combined attributes of the two aforementioned CDC best practices for HIV prevention for women to develop the Women’s PrEP Project (W-PrEP), a clinic-level, sociostructural intervention. We designed W-PrEP to provide education, resources, and support to empower and enable sexual and reproductive health clinics to provide high-quality, nonstigmatizing, universal PrEP services to women with increased exposure to HIV, in particular Black women of reproductive age. This paper describes the formative work adapting and developing the W-PrEP intervention from the CDC best practices reviewed earlier.

## Methods

We conducted a formative study for the intervention adaptation from November 2021 to October 2023, including marked expansion of existing training materials and toolkits.

### Ethical Considerations

We received institutional review board approval from the MedStar Health Research Institute Institutional Review Board (#0006908). We obtained written or electronic informed consent from all participants for participation in interviews and focus groups and the recording of these sessions. Participants were compensated with a US $100 gift card for completion of interviews or focus groups. All study data were stored behind a firewall and deidentified before qualitative analysis.

### Intervention Development Process Model

We used the Assessment, Decision, Adaptation, Production, Topical experts, Integration, Training, and Testing (ADAPT-ITT) model as a framework to develop a culturally appropriate and tailored EBI [53]. ADAPT-ITT has been used to successfully adapt a host of diverse HIV prevention interventions to meet the needs of a new intended population or setting [54-57]. We used the Project Shikamana EBI and the HIV PrEP Services for Urban Women EII as the base cases to initiate the intervention development process (Table 1). We focused on sociostructural factors that women in the region indicated were barriers to and facilitators of engagement in the PrEP cascade (ie, stigma and discrimination related to HIV and PrEP within health care settings, provider sensitization, and PrEP navigation and support). Using ADAPT-ITT, we built upon those findings and used multiple qualitative methods, such as in-depth interviews and focus group discussions, to refine the existing interventions (Table 2). This paper describes the first 6 steps in the model in preparation for pilot testing.

**Table 1:** Base case interventions.

Intervention	Description
Project Shikamana EBI^a^ [50-52]	Peer navigation and education: Project Shikamana offered both venue-based peer education, which included condom distribution and HIV counseling and testing, and peer service navigation for women living with HIV, including social support to promote HIV treatment access and adherence. Provider sensitivity training: Trainings for HIV health care providers promoted nonstigmatizing, quality health services and were conducted in coordination with peer navigators. Community-led drop-in center: The drop-in center was designed to stimulate social cohesion and mobilization activities and offered HIV counseling, testing, and condoms. The center hosted support groups for community members and convened seminars on topics such as stigma, gender-based violence, income generation and savings groups and HIV prevention.
HIV PrEP Services for Urban Women EII^b^ [49,50]	Structured clinic-wide training and refresher trainings: Trainings emphasized universal provision of PrEP^c^ education and counseling and prescribing PrEP, including screening laboratory analyses and techniques to encourage PrEP adherence and persistence. Trainings included sample language for counseling and role-plays. Provider toolkit: The toolkit featured an EHR^d^ PrEP screening prompt, which inquired if patients were interested in discussing starting PrEP as part of their visit, along with screening questions regarding indications for PrEP. The toolkit also offered ID badge card–sized reference “cheat” sheets and workstation reference sheets with indicated laboratory testing, dosing, and follow-up for PrEP and nPEP^e^ initiation. Looped patient-facing PrEP educational videos: The intervention offered looping, short educational videos about PrEP and sexual health in the waiting room, with the goal of increasing PrEP awareness and normalizing PrEP to facilitate conversations with health care providers.

^a^EBI: evidence-based intervention.

^b^EII: evidence-informed intervention.

^c^PrEP: pre-exposure prophylaxis.

^d^EHR: electronic health record.

^e^nPEP: nonoccupational postexposure prophylaxis.

**Table 2:** Study activities and measures by Assessment, Decision, Adaptation, Production, Topical experts, Integration, Training, and Testing (ADAPT-ITT) phase.

ADAPT-ITT phase [53]	Activities and measures
*Assessment:* obtain a comprehensive understanding of the target population	Formation and initial meetings with the joint community and scientific advisory committee, consisting of a social justice advocate, an HIV and HIV prevention advocate, the founder of a nonprofit organization focused on HIV and faith communities, a family planning specialist and reproductive rights advocate, a family physician with expertise in provider education and HIV prevention, and a nurse practitioner with expertise in HIV prevention and implementation science Key informant interviews with an executive director of the national office of an international women’s HIV organization, an executive director of a local women’s HIV and HIV prevention community-based organization, a director of clinical services of a local branch of a national reproductive health care service provider, a director of a government sponsored sexual health clinic, a director of women’s health at a local HIV health center, a coordinator of a government-sponsored PrEP^a^ campaign for women, an HIV prevention coordinator at a local federally qualified community health center, a family medicine provider with a focus on HIV and STI^b^ prevention, a family-nurse practitioner working in HIV treatment and prevention, a social worker at a preventive health clinic focused on female sex workers (n=10) Focus group discussions with health care providers with (n=1 group) and without (n=1 group) experience prescribing PrEP, PrEP navigators and educators (n=1 group), and Black women of reproductive age with (n=2 groups) and without (n=2 groups) previous PrEP experience
*Decision:* select interventions and decide whether to adopt or adapt	Review of CDCc Best Practice base cases (ie, Project Shikamana EBI^d^ and the HIV PrEP Services for Urban Women EII^e^) Review of current literature on PrEP and HIV prevention–related interventions for women
*Adaptation:* use a pretested methodology to better understand how to adapt	Theater testing of a mock-up of the intervention flow, clinic-wide training materials, and toolkit with the participants from the health care provider focus groups
*Production:* create an adaptation plan and determine goals	Production of the initial prototype, including adaptation or development of the following: (1) clinic-level PrEP rollout toolkit, clinical team toolkits, and PrEP navigator toolkit; clinic-wide training and PrEP navigator training; increased availability and accessibility of PrEP services; PrEP navigation program, including delineation of the role of the navigator; and patient-facing PrEP educational resources
*Topical experts:* provide substantive content and technical assistance	Engagement of the community and scientific advisory committee to elicit feedback on the intervention prototype
*Integration:* integrate all forms of information	Reflect feedback from the advisory committee into the refined intervention prototype

^a^PrEP: pre-exposure prophylaxis.

^b^STI: sexually transmitted infection.

^c^CDC: Centers for Disease Control and Prevention.

^d^EBI: evidence-based intervention.

^e^EII: evidence-informed intervention.

### Study Setting

Washington, DC is an epicenter of the US HIV epidemic. At the time of the intervention refinement, the prevalence of HIV among women in Washington, DC was 1209 per 100,000, nearly 7-fold the national average (170 per 100,000) [58]; the prevalence among all women was 0.9%, but nearly double (1.7%) among Black women [58]. At the time of this study, Black women represented 1 in 4 new HIV diagnoses in Washington, DC and 90% of new diagnoses among women [58]. This formative study was conducted online by the research institute associated with an inner-city tertiary care center. Given the timing during the COVID-19 pandemic and to increase access for participants who would be unable to attend in person, all qualitative research was conducted remotely via a secure Microsoft Teams platform.

### Qualitative Tools and Data Collection

We conducted semistructured in-depth interviews with key informants as part of the *Assessment* phase. We sought key informants with intimate knowledge and understanding of barriers to and facilitators of PrEP among women, with a focus on individual, social, and structural factors. We identified key informants from local community-based organizations, hospitals, clinics, and HIV prevention organizations through referrals from the Washington, DC. Center for AIDS Research; our joint community and scientific advisory board; and clinical and community professional relationships of the research team. Target recruitment was 10 key informant interviews, and key informants were invited via email by the principal investigator to participate. The key informant interviews focused upon (1) factors influencing PrEP use among women and (2) intervention components key to improving equitable provision of PrEP on a clinic level as well as complementary tools and materials that may support community needs and priorities. Accordingly, interview questions focused on (1) sociostructural barriers to and facilitators of PrEP use among Black women of reproductive age and (2) assessment of the model components for adaptation. Salient themes and questions arising from the key informant interviews informed the focus group discussion guides.

We also conducted 7 focus group discussions, separating participants into groups of (1) health care providers with experience prescribing PrEP; (2) health care providers without experience prescribing PrEP; (3) PrEP clinical navigators, peer navigators, and educators; (4) Black women of reproductive age with experience taking PrEP (n=2 groups); and (5) Black women of reproductive age without previous experience taking PrEP (n=2 groups). Providers, navigators and educators, and patients were recruited from medical establishments (eg, hospital-based clinics and community health centers) throughout Washington, DC. We invited women’s health providers from varying fields (eg, obstetrics and gynecology, family medicine, and pediatrics), institution types (eg, free-standing reproductive health center, government-sponsored sexual health center, tertiary care center, and federally qualified community health center), and medical training types (ie, physician, nurse practitioner, and physician’s assistant) to participate via email. PrEP navigators and educators from differing institution types were also invited to participate via email. A formal email invitation to participate in interviews was sent by the principal investigator, and a research coordinator then followed up to assist with scheduling. Patient participants were identified from the women’s health clinics and the electronic health record (EHR) and were recruited and scheduled by research coordinators via phone.

Health care provider focus group guides concentrated on the proposed PrEP training materials and format, identification of key toolkit resources to provide PrEP services and integration of PrEP into sexual and reproductive health care, and inclusion of a PrEP navigator and educator in the clinic setting. Navigator and educator focus group guides centered on the roles and characteristics of the PrEP navigator as well as training and resources. Patient focus group guides explored women’s health priorities and barriers to preventive health, such as PrEP and ideal provision of PrEP services, including education and access, PrEP navigation and the key characteristics and roles of a PrEP navigator and educator, and social support.

We conducted theater testing of a mock-up of the adapted training and planned intervention and implementation flowchart among these same health care providers in 2 follow-up focus group discussions. Providers offered feedback on the content and delivery of Microsoft PowerPoint training modules and the multipronged intervention.

### Qualitative Analysis

We audio recorded interviews and focus groups with the consent of participants, which were then transcribed verbatim. We applied rapid qualitative analysis [59-61] to systematically use qualitative feedback from the interviews and focus groups to refine the intervention. To this end, we developed summary templates to identify the coder, respondent role, and coding domain (ie, strengths, information gaps, and actionable improvements). We conducted analyses of a subset of interviews (ie, 1 from each respondent type) to ensure adequate fit of the template to the data, and then we coded the remaining transcripts. For the formative ADAPT-ITT phases (ie, *assessment* through *integration*), we developed and populated matrices to identify salient themes that represented topics, information, or activities to include in the adapted intervention. For the *training* and *testing* ADAPT-ITT phases, we developed and populated matrices to align with germane components of the practical, robust, implementation, and sustainability model implementation framework. These summaries guided team discussions and W-PrEP intervention refinement.

## Results

### Assessment Phase

#### Joint Community and Scientific Advisory Committee

In this first phase of ADAPT-ITT, we convened a joint community and scientific advisory committee (Table 2). In the committee’s initial meeting, the research team provided an overview of the existing research, EII, and EBIs for women as the basis for discussion of the gaps in HIV prevention for women and opportunities for HIV prevention interventions. The committee discussion supported the focus of the key informant interview guide on sociostructural barriers to facilitators of PrEP for women and assessment of base case components for adaptation.

#### Key Informant Interviews

We completed 10 semistructured interviews with community-based organization leadership, clinic and HIV prevention operation managers and leadership, health care providers, social workers, and educators and coordinators. Table 2 describes participants. Illustrative quotations from the key informant interviews (and from the subsequent qualitative portions of the study) are presented in Multimedia Appendix 1. Key informants identified several key barriers to PrEP use among Black women of reproductive age. The most commonly cited barrier was the lack of availability and accessibility of PrEP education and services for women, including the lack of integrated HIV prevention and PrEP services into primary women’s health care, the challenging logistics of navigating PrEP care in light of competing priorities and social determinants of health, and the lack of culturally appropriate PrEP marketing and educational materials for women. Suggested opportunities included increased provider education; integration of PrEP into women’s primary and reproductive and sexual health care; and use of the full clinical team, including PrEP navigators, serving as PrEP educators and ambassadors. Key informants additionally identified low perceived risk of HIV acquisition and low awareness of PrEP as critical barriers for women. Many referenced the dearth of public health and industry marketing campaigns focused upon women (compared to men who have sex with men). Multiple key informants noted continued stigma related to taking a medication associated with sexual “promiscuity,” men who have sex with men, and HIV and barriers to disclosure of PrEP use to support networks, including partners and family members.

We queried key informants about the adaptation of Project Shikamana and the HIV PrEP Services for Urban Women as well as key components to retain. There was universal enthusiasm for the inclusion of a PrEP navigator. Many expressed that PrEP navigators should be from the community and reflect the intended demographic (ie, Black women of reproductive age). There was a sentiment that PrEP navigators should serve as representatives of the community in the clinical setting as a means of combating medical mistrust and misconceptions surrounding PrEP. Participants suggested that PrEP navigators could also identify community needs and assets surrounding PrEP and HIV prevention. Many participants also identified the importance of the navigators in PrEP education and in navigation of PrEP services, including insurance prior authorization, appointments, and prescription assistance programs for uninsured or underinsured patients.

In terms of PrEP training for the clinic team, key themes included centering the community and patient voice in the training, promoting sex-positive language (versus risk-based language) as a nonstigmatizing way of conversing about PrEP and HIV prevention, and the importance of bias training (to reduce racial, sex, and gender biases).

Although key informants underscored the importance of community support in engagement and retention in the PrEP cascade for women, they emphasized harnessing the power of existing community-based organizations and referring to these organizations while using intervention resources to create a supportive, safe, stigma-free environment to learn about PrEP and access PrEP within the clinic.

#### Focus Group Discussions

Through the key informant interviews, we identified tools, strategies, and experiences that the intervention should address. In the next phase, we conducted focus groups to gain insights into how to develop the program content. We conducted 3 sets of focus group discussions separately with providers, PrEP navigators and educators, and patients, specifically Black women of reproductive age (4-8 participants per group).

#### Focus Group Discussions: Health Care Providers

A total of 10 health care providers participated in 2 focus group discussions: one group with 6 providers with PrEP experience and another group with 4 providers without PrEP experience. Providers across both groups agreed upon the utility of workflow aids, such as EHR order sets, PrEP reference “cheat” sheets, and badge cards or “badge buddies” to facilitate incorporation of PrEP care into their clinical practices. There was also a suggestion for a clinic PrEP champion as a resource to assist with complicated clinical scenarios. Providers agreed that telehealth was helpful for PrEP continuation, especially for patients with competing priorities or social determinants of health (eg, transportation and limited time off from work) that may limit frequent in-person visits. Providers were enthusiastic about the potential for a PrEP navigator. There was consensus that PrEP clinical navigation (eg, obtaining insurance prior authorization and assisting with rescheduling appointments) was logistically key to integrating PrEP into a bustling clinical practice with overstretched providers. In addition, they supported a PrEP navigator serving to introduce PrEP to patients and provide additional education about PrEP to alleviate some of the logistical time constraints on patient visits. Providers prioritized clinical navigation and education services (over social support) to operably integrate PrEP services into their clinical practice. Several providers reasoned that social support issues outside of the scope of the clinical PrEP navigator could be referred to the outpatient social work team.

Providers preferred in-person trainings (to online or hybrid), with an asynchronous alternative for rotating learners or review at a later time point. Multiple participants emphasized the importance of refresher trainings several months after the initial training. There was a call for interactive training activities (eg, role-playing) both to keep participants engaged and to enact new skills. In addition to education on HIV prevention and PrEP, providers requested inclusion of practical tips and logistics and sample scripts for incorporation of PrEP services into their clinical practice in a nonstigmatizing way. In addition, providers requested the incorporation of the epidemiologic context for HIV prevention among women and specifically requested centering the community and patient voice and perspective throughout the trainings.

#### Focus Group Discussions: Navigators and Educators

PrEP peer and clinical navigators and educators (n=4) participated in the focus group. The group agreed that the ability to connect quickly with patients was more important than shared background or personal experience with PrEP. They emphasized the importance of navigation to find room in a patient’s lifestyle for PrEP (in the face of competing priorities) but also mentioned the importance of social and emotional support and referral to social services.

The group preferred in-person training and supported a supplemental asynchronous option. They shared the providers’ preference for interactive trainings with role-playing. In addition to the basics of HIV prevention and PrEP, navigators and educators identified key training topics of social and structural determinants of health, advocacy skills, and sexual and reproductive health.

#### Focus Group Discussions: Patients

A total of 16 patients (n=8 with experience taking PrEP and n=8 without experience taking PrEP) participated in 4 focus group discussions, divided by previous PrEP experience. Preventive health (eg, nutrition, physical activity, mental health, and routine health screenings) was identified as a common health goal. Patients described provider bias surrounding sexual health, costs associated with navigating the health system and with accessing healthy foods and fitness options, and competing priorities as barriers to meeting their personal health goals.

Nearly all patient participants were enthusiastic about culturally appropriate educational videos about PrEP to increase PrEP awareness in the waiting room; there was also interest in women-centered PrEP pamphlets, posters, and links to websites with additional information about PrEP. Although there was no consensus on who (eg, nurse, health care provider, or PrEP navigator) should introduce PrEP, there was agreement that what mattered most was that PrEP was introduced in a universal, nonstigmatizing manner.

There was agreement that the PrEP navigator should be a cisgender woman who shares their cultural and community background (ie, Black and familiar with or from the locality), ideally someone who is currently taking PrEP or has taken it in the past. There was a strong appeal in being able to discuss PrEP with a trusted individual having similar life experiences and first-hand experience with PrEP yet outside of the medical field. Participants voiced that the roles of educators, prevention navigators, and supportive allies in the process were all important.

There was little consensus on the ideal setting or mechanism for social support, given that participants accessed and used social support systems in different ways. Preferences were split between in-person and online support mechanisms. Several supported an online option that could provide flexibility and continuity for women with competing priorities. Those who preferred an in-person support setting were divided between pop-up events versus a permanent community support setting; those who favored a permanent space were further conflicted on whether the space should be associated with the hospital or embedded in the community.

### Decision

In preparation for the *decision* phase, the research team reviewed the findings of the key informant interview and focus group discussion rapid qualitative analyses and met with the joint advisory board and 5 additional national HIV prevention intervention experts to discuss existing resources and planned intervention strategies. The research team also updated a scoping review of the contemporary relevant literature. The research team reaffirmed the plan to adapt and build upon 2 base cases (Table 2), using the multipronged, sociostructural approach of Project Shikamana and the clinic-centered intervention setting and educational focus of the HIV PrEP Services for Urban Women. Notably, given the feedback from the key informant interviews on harnessing existing community support resources and the lack of consensus on social and community support mechanisms, the research team decided to focus on the “provider sensitivity training” and “education and navigation and support” components of Project Shikamana, specifically creating a nonstigmatizing, supportive, and safe environment for the provision of HIV prevention education and services within the clinic setting (with referrals to outside organizations) rather than creating an intervention-specific community support component. On the basis of our extensive background research and our findings in this formative work, the research team affirmed the decision to use the socioecological model given the multipronged and multilevel approach of the intervention [29,62,63].

### Adaptation

Provider focus group participants were invited back for theater testing of the planned intervention flowchart and a mock-up of the adapted trainings and toolkits, expanding on the HIV PrEP Services for Urban Women and Project Shikamana’s provider sensitivity training, incorporating lessons learned from the *assessment* phase. We elicited topic-specific feedback on length, level of detail, and preferred formats and mediums for learning and skill enactment within the training modules as well as feedback on specific slides. In addition, we encouraged suggestions from participants to improve or refine the toolkits and intervention flowchart. Participants emphasized the overall approval of the intervention mock-up and provided detailed feedback to improve clarity and incorporate additional resources or tools, such as a badge card with reminders about prescribing requirements.

### Production

Incorporating the theater testing feedback, we fleshed out a prototype for each of the components of the multipronged intervention; Table 3 outlines the intervention components. We adapted and expanded upon the clinic-wide trainings, incorporating more role-play and segments with the patient perspective taken from previous qualitative work [30], as well as identified key topics, such as how to incorporate PrEP care into clinical practice. We curated a PrEP clinical navigator training program using materials from the Please PrEP Me navigator training while incorporating feedback from qualitative data collection [64-66]. In addition, we collated educational resources for the clinic team, PrEP navigators, and patients, including a patient-facing educational website. We updated and developed a variety of toolkits for the clinic-level rollout of W-PrEP and rollout of long-acting injectable PrEP as well as role-specific toolkits for the clinic team members and the PrEP navigator. We also created a PrEP clinical navigation flowchart, with multiple options for modification and adaptation depending on the availability of clinical space and when patients had downtime between clinical activities (eg, triage by medical assistant and evaluation by health care provider).

**Table 3:** Adapted Women’s PrEP Project (W-PrEP) intervention components.

Component	Description
Clinic-level PrEP^a^ rollout toolkit	W-PrEP implementation checklist Algorithm for universal PrEP counseling and services Implementation algorithms for rollout of new PrEP formulations (including prior authorization) PrEP navigator job description Identification of a PrEP champion
PrEP champion	Health care provider to serve as a PrEP expert and site PrEP champion Responsibilities include facilitation of trainings and consultation on both clinic-level implementation of PrEP services and complex clinical cases
Clinic-wide training	Key training topics include the following: HIV testing (which test at which time) and STI^b^ testing; taking a patient-centered sexual and social history; positive reframing of prevention communication and counseling around PrEP, how to incorporate patient-centered PrEP and nPEP^c^ provision for women into routine care; PrEP initiation, continuation and persistence, and discontinuation; nPEP initiation and transition to PrEP; implicit and explicit bias training; trauma-informed care; epidemiology of the US HIV epidemic; and social and structural determinants of health related to HIV Key features are as follows: synchronous, interactive Microsoft PowerPoint presentations scripted for reproducibility and scalability; asynchronous prerecorded modules to accommodate irregular schedules of health care providers and rotating learners, with activities to engage the asynchronous individual learner; and centering of the patient voice and experience throughout both modules
Increased availability and accessibility of PrEP follow-up	Increased availability of telehealth and web-based medical visits for PrEP follow-up visits: These visits are designed as alternatives to in-person visits for women who were not ready to start PrEP but wanted to learn more about PrEP (from providers and PrEP navigators) or for women who have been prescribed or started PrEP to follow up regarding issues with prescription pickup, medication side effects, adherence, or other concerns. Off-site laboratory referrals: Providers will offer referrals to off-site commercial laboratories for follow-up STI and HIV testing if more convenient “PrEP talk” with PrEP navigator: Email, text, and telephone follow-up options will be offered to address concerns and offer education.
Clinic team toolkit	EHR^d^ PrEP order set (eg, STI screening laboratory orders, PrEP injection or prescription orders, and HIV testing orders) EHR PrEP quick text to facilitate documentation of PrEP education and counseling and prescription and PrEP EHR prompts to remind and encourage providers to counsel all patients about PrEP (dependent upon EHR settings) PrEP and nPEP badge buddy: an ID badge card–sized PrEP and nPEP reference “cheat” sheet with recommended STI and HIV screening, dosing information, and follow-up PrEP and nPEP reference “cheat” sheet and checklists: full-size reference sheet and PrEP start and continuation checklists for quick reference during a busy clinic day (recommended to be posted at the provider workstation) Sample scripts for introduction to PrEP (for medical assistants and nurses) and sample scripts for introduction and counseling for prescribing providers “Ask me about PrEP!” swag (eg, pins and lanyards)
PrEP navigator training	In addition to the clinic-wide PrEP training, through a combination of tailored one-on-one training from a PrEP education and navigation expert, independent learning, and online modules, the PrEP navigator will receive training in the following: PrEP navigation through the Please PrEP Me Navigator training [64], implicit biases in the health care setting [66], trauma-informed care [65], and introduction to reproductive and sexual health topics (led by a site health care provider and topically tailored to the clinical context)
PrEP navigator toolkit	In addition to the aforementioned clinical tools, the PrEP navigators will have access to the following: PrEP quick text to facilitate documentation of PrEP navigator education and counseling, including assessment of barriers to PrEP; sample script for introduction to PrEP and counseling about PrEP; and patient-facing educational resources
PrEP navigation and support services	Informed by our formative work, PrEP navigators will be cisgender women, ideally from the same community the clinic serves and with first-hand experience with ARVs^f^ through PrEP and HIV. PrEP navigators will provide the following: PrEP educational services—introducing PrEP to patients, combating misperceptions or myths about PrEP; PrEP social and adherence support—breaking down social barriers to PrEP and mitigating the internalization of PrEP-related stigma and building and encouraging individual agency or self-efficacy to initiate and adhere to PrEP; and PrEP navigation services—facilitating access to PrEP appointments, assisting with insurance authorization, and referrals to indicated social services (eg, substance use, gender-based violence, and mental health)
Patient-facing PrEP resources	Videos on PrEP and women’s health and sexual health topics (selected by the clinical site) will be spliced and played on loop in the clinic waiting room to increase awareness of PrEP Inclusive pamphlets on PrEP in the waiting room and examination rooms Inclusive posters about PrEP and HIV prevention displayed in the waiting room and examination rooms W-PrEP educational website QR code available in the waiting area and distributed by the PrEP navigator

^a^PrEP: pre-exposure prophylaxis.

^b^STI: sexually transmitted infection.

^c^nPEP: nonoccupational postexposure prophylaxis.

^d^EHR: electronic health record.

^f^ARV: antiretrovirals.

### Topical Experts and Integration

We engaged the community and scientific advisory committee as *topical experts* to review the prototypes developed in the *productio*n phase (ie, the intervention flowchart, toolkits, training modules, and educational resources). The committee expressed enthusiastic support for the adapted intervention and offered minimal feedback to incorporate into the intervention components.

## Discussion

### Principal Findings

Using the ADAPT-ITT model, we iteratively adapted two CDC best practices in HIV prevention, using the sociostructural approach of Project Shikamana and building from the training model of the HIV PrEP Services for Urban Women. We found that while the core elements of provider sensitivity training, navigation, and support of Project Shikamana were maintained, the urban mid-Atlantic context lent itself better to a clinic-based approach. Thus, we focused on a supportive, trauma-informed clinical environment without a separate community support venue. In addition, the navigation model shifted from a community peer support model to a PrEP navigator supporting clinic workflows to meet the needs of a bustling urban women’s health clinic with a high patient volume. The training from the HIV PrEP Services for Urban Women was significantly expanded to center the patient voice and prioritize shared decision-making throughout the modules. We incorporated group activities, discussions, and role-plays to make the training more interactive. Added content included a focus on social and structural determinants of health, trauma-informed care, and incorporation of PrEP counseling into clinical practice. The adapted W-PrEP intervention features clinic-level modular trainings; a toolkit and resources for clinic-level implementation of PrEP services; toolkits and resources for provider and clinic team members; a clinical PrEP navigation program, including training and resources; and patient-facing woman-centric educational and support resources, including a website focused on PrEP for women, all to be tested in the pilot *testing* phase.

### Comparison With Prior Work

At the time of intervention development, Project Shikamana and the HIV PrEP Services for Urban Women were the only two best practices in the CDC compendium addressing HIV prevention for women. W-PrEP is now among a growing number of interventions published in the last several years intended to increase engagement and retention in the PrEP cascade among women, several specifically responsive to Black women [67-71]. Similar to the POWER Up intervention [70], developed in parallel with W-PrEP and currently under evaluation, W-PrEP uses a sociostructural approach. As a clinic-level intervention, W-PrEP expands upon our previous work [49,72] to more fully address identified patient-, provider-, and clinic-level socioecological barriers, such as low awareness among patients, low knowledge and confidence in how to provide PrEP among providers, and clinic-level challenges of systems and logistics for obtaining prior authorization and tracking patient follow-up.

### Limitations

W-PrEP was adapted in an urban, mid-Atlantic setting and is specifically responsive to the barriers to PrEP for Black women. Although we posit that our developed intervention is likely generalizable to women with increased potential exposure to HIV throughout the United States, we recognize that there may be cultural differences specific to geographic regions. We catered the intervention development to sexual and reproductive health providers, given the natural synergy between HIV prevention and sexual and reproductive health and the patient populations that these providers care for. We again recognize that this intervention may need additional adaptation for implementation in the primary care setting, including additional modules for providers who also care for patients assigned male sex at birth.

### Conclusions

This paper details the adaptation process of two CDC best practices to create a program that is responsive to the needs and experiences of the various groups involved in its delivery. We provide such details because the practicalities and logistics of adaptation are often unarticulated and completed behind a curtain. We sought to provide insights into the adaptation process from the ground level. The adaptation of the W-PrEP program integrated key elements of the local context for women with potential exposure to HIV as well as health care providers, clinic staff, and PrEP navigators. Next steps include piloting the adapted intervention in a real-world setting for feasibility, acceptability, and preliminary efficacy before launching a multisite trial to assess impact on the PrEP cascade.

## Appendix

Multimedia Appendix 1 Example quotations by Assessment, Decision, Adaptation, Production, Topical experts, Integration, Training, and Testing (ADAPT-ITT) phase and activity.

## Abbreviations

ADAPT-ITT: Assessment, Decision, Adaptation, Production, Topical experts, Integration, Training, and Testing
CDC: Centers for Disease Control and Prevention
EBI: evidence-based intervention
EHR: electronic health record
EII: evidence-informed intervention
PrEP: pre-exposure prophylaxis
W-PrEP: Women’s PrEP Project

## Data Availability

The datasets generated or analyzed during this study are available from the corresponding author on reasonable request.
