# Supplementary material for: Development of a Clinic-Based, Sociostructural Intervention to Improve the Provision of Pre-Exposure Prophylaxis for Cisgender Women: Formative Study Using the Assessment, Decision, Adaptation, Production, Topical Experts, Integration, Training, and Testing (ADAPT-ITT) Framework
Source: JMIR Form Res. 2025 Aug 6;9:e75922. doi: 10.2196/75922 (PMC12368467; doi:10.2196/75922)
Supplement: Multimedia Appendix 1 [file formative_v9i1e75922_app1.docx]

**Multimedia Appendix 1.** Exemplary quotations by *assessment, decision, adaptation, production, topical experts, integration, training, and testing* (ADAPT-ITT) phase and activity

| ADAPT-ITT phase and activity | | Quotations |
| --- | --- | --- |
|  | *Barriers to PrEP*^b^*: accessibility and availability* | - “[A] lot of...the providers are not talking about PrEP, they are not well versed in PrEP.” - KI 6: Black cisgender woman aged 26 y, prevention coordinator at a community health center |
|  | *Barriers to PrEP: accessibility and availability* | - “One of the other barriers is just medication access. So, a lot of them, when we send the prescription to the pharmacy, I have to do close follow-up to make sure that they actually pick up the medication. Sometimes, if they go to the pharmacy, if there is an insurance issue, a lot of times it’s a deterrent for them continuing with the process of initiating the medication. So, when I see them, I do very close follow-up. I try to stay in touch to make sure that they have picked up the medication, and to check in to make sure that they’ve started and just to assess if they’re having any side effects and things like that.” - KI 8: Black cisgender woman aged 33 y, family nurse practitioner specializing in HIV and HIV prevention |
|  | *Barriers to PrEP: health literacy and perceived risk* | - “[Women] do not see their lives as at-risk. …They do not understand…high levels of incarceration in the community, or transactional sex work…as a problem, …they see that just as regular life in their communities. And then if they were asked by the provider, do you engage in any high-risk activities, they’re obviously going to say no, because they don’t consider the activities that they do as high risk…Sometimes when you share, or describe some…STDs or things like that that women experience, they will say, oh my gosh, I’ve had that. Or I’ve experienced that. And they didn’t know what it was.” - KI 1: Black cisgender woman aged 47 y, regional leader of an international HIV and HIV prevention advocacy organization |
|  | *Barriers to PrEP: health literacy and perceived risk* | - “I think the main barrier is lack of perceived risk. Most women don’t believe or know that they’re at risk for acquiring HIV. And many of them have not even heard of PrEP before. So, lack of education as well. …When people hear the word PrEP they think about the [men who have sex with men (MSM)] community. So, they don’t even know that the medication has been approved for female use. So, like I said, the biggest issue is lack of education.” - KI 8: Black cisgender woman aged 33 y, family nurse practitioner specializing in HIV and HIV prevention |
|  | *Barriers to PrEP: stigma* | - “When we talk to MSM and trans women about PrEP, there is less of a stigma associated with being on PrEP. In some ways, it’s socially acceptable and it’s socially desirable. So, on dating apps for instance, it’s not uncommon for MSM or trans women to say, ‘HIV-negative, disease and drug-free, on PrEP.’ It’s almost like a badge of honor or something. But with ciswomen, when we talk to them about PrEP, we get a little bit of like, ‘What are you trying to say? Why do you think I need this medication? What is that saying about me? What are you saying about my partners?’ And so, that’s also a barrier, the perception of what it means to be on PrEP, and the stigma associated with HIV.”- KI 2: White (non-Hispanic) cisgender man aged 50 y, Department of Health interim division chief for STI^c^ and Tuberculosis Control and sexual health clinic health care provider |
|  | *Model adaptation: PrEP navigation* | - “[I]t addresses the medical mistrust piece because you’ve got a trusted messenger there who’s gonna have evidence-based information, but can deliver in a way that maybe will resonate more, so I think that’s great. I also think that sometimes, it’s helpful to see someone who looks like you, that you feel like you can relate to, and who can also—like I said—take you by the hand to go through that process.” - KI 5: Black cisgender woman aged 32 y, manager of women’s wellness programs at a local HIV-focused organization |
|  | *Model adaptation: training* | - “Definitely…history…helping physicians understand the reasons behind medical mistrust in communities of color—the historical context. ...I would definitely want a conversation around taking a sexual history, having these difficult conversations around sexual health, how to have them appropriately, maybe something specific…to the experiences of Black women.” - KI 5: Black cisgender woman aged 32 y, manager of women’s wellness programs at a local HIV-focused organization |
|  | *Model adaptation: training* | - “A baseline conversation about what PrEP is, the importance of adherence, how the medication works, that it’s important that it’s taken every day to be most effective, typical side effects, and maybe a conversation about routine follow-up and how often they would need to be seen and monitored, and lab testing. …[T]hey definitely should be able to have a conversation with [patients] about just the overall rates of HIV within this area so that they get a better understanding of their risk and why they’re even bringing PrEP up as a consideration for them. …[T]he conversation should definitely lean more towards prevention and holistic care. You’re sexually active and taking this medication is a part of ensuring that you’re safe and you have safe sexual practices and it can alleviate some of the fear that you may have around acquiring HIV. So, not necessarily saying because you have several partners you’re considered high risk, but shifting the conversation towards we know that you’re sexually active. You don’t always use condoms. As a part of your holistic care, it’s a good idea to consider taking this medication as an additional layer of protection for yourself.” - KI 8: Black cisgender woman aged 33 y, family nurse practitioner specializing in HIV and HIV prevention. |
|  | *Model adaptation: community support* | - “[T]here [are] a lot of [community-based organizations (CBOs)] that are currently out there doing a lot of really, really great work…you [will] have more impact by leveraging existing relationships [with] existing CBOs that already have community buy-in, and [a]re…more grass roots.” - KI 3: White cisgender man aged 37 y, family medicine practitioner |
|  | *Key components of the intervention: PrEP navigation* | - “[W]hen we first initiated PrEP at my clinic, we actually had an education coordinator who had reached out to each person after they started PrEP, just to follow up and see were they able to fill their prescription, do they have any issues or anything that we need to assist them with. And so that was really helpful in kind of identifying issues. We don’t have that anymore now. And so now we are kind of seeing some challenges where people are paying out of pocket for their medications, you know, things that we didn’t realize at their initial appointment that we’re learning later on.” [Provider participant 5] - “I would love for us to have nurses or a navigator, somebody to provide education.” [Provider participant 6] |
|  | *Key components of the intervention: toolkit* | - *On badge cards and reference sheets*: “Something to be able to refer back…that is handy that can either be kept on you…[or] where people sit and are writing notes. …[H]aving that sort of quick start PrEP guide…right next to the desk of the attending physician…is really helpful, because otherwise I would forget exactly what I’m supposed to order, what to do, who to screen, how to screen or remember to screen.” [Provider participant 4] - *On sample scripts*: “[T]echniques of how to frame the conversation and maybe suggested talking points or, you know, just some like suggested language to use of how to bring up the topic and what are ways to just start the conversation that are recognizing like our patients, backgrounds and experiences and maybe that don’t sound judgmental.” [Provider participant 6] - *On EHR*^e^ *tools*: “We use…a [EHR quick-text tool] to say…PrEP was discussed with this patient…what their risk may be and…what we what was discussed during the visit. So anyone that was at risk or…[had] identified risk factors we would put that [quick text] in for all the patients and do that use the HIV counseling, ICD 10 code. …And order sets for initiation of PrEP too, for the labs, can be really helpful and that might take just one step of complexity off the table to make it a little bit easier for providers to have more time to discuss it and less time ordering labs.” [Provider participant 5] |
|  | *Key components of the intervention: PrEP champion* | - *On a PrEP Champion*: “Having a champion…[S]omeone that a new PrEP provider could call…when [the patient’s] …labs come back and their LFTs are abnormal. And so [instead of] just giving up on PrEP, which I think a lot of people would do, having someone that they can call.” [Provider participant 2] |
|  | *Key components of the intervention: clinic-wide training (content and format)* | - *On training on integration into clinical practice:* “[T]his is how a general OBGYN can effectively start prescribing PrEP to their patients and protect them from HIV. It’s like very practical. You attend this session; you will be confident to do this. Not like you learned a little bit about it and now you sort of aware this exists and other people do it, but this is how you can do it in your practice.” [Provider participant 6] - *On format*: “Some of the most effective and enjoyable trainings are small group sessions…like the simulation lab, where you may work in a small group and have a leader or teacher that presents material, maybe goes through cases or situations and it’s interactive or hands on in some way.” [Provider participant 6] - *On format*: “Case studies, especially in these types of situations, would be really helpful to kind of go through the different patient types that might or might not be candidates for PrEP. Or you know whether there are specific patients with contraindications for PrEP and talking about that. I think as opposed to a list, it’s helpful to kind of relate it to a real person or a patient. I think that also, that plus written materials is helpful.” [Provider participant 4] |
|  | *Characteristics and role of the PrEP navigator* | - *On characteristics:* “[P]ersonability. Being able to communicate and relate to the patients in the specific area where you work, you know, you want to be able to cater to…the people surrounding you that’s going to come into your clinic.” [Educator and navigator participant 5] - *On characteristics:* “[It’s] not…a big issue whether it’s a woman or man, it’s whether or not you are communicating the PrEP information properly.” [Educator and navigator participant 2] - *On role:* “Informing them about PrEP, addressing any questions that the patient may have about PrEP. Possibly, if the facility does not have a specific person who is responsible for navigating the patient through the insurance process, then that navigator would actually assist the patient with navigating the insurance process and also be that person who kind of like checks up on them to say, hey, how’s it going with your PrEP? Have you…picked up a routine with taking your PrEP? Do you need assistance with figuring out how to make this a part of your lifestyle? I think that’s what a PrEP navigator should actually be responsible for.” [Educator and navigator participant 2] |
|  | *Barriers to health priorities* | - *On accessibility*: “I will agree on financial burdens and accessibility. …If you live in a certain part of town, you may not have access to different resources that someone else has…If someone doesn’t have a form of transportation, things like that. …I see that a lot…in my community.” [Patient participant 11] - *On competing priorities*: “It’s like I have to choose one of the healthy things, but I can’t do them all.” [Patient participant 8] - *On competing priorities*: “I would say navigating the healthcare system…So if you have different chronic illnesses, …you’re gonna see a GI versus your allergist versus your primary care.” [Patient participant 7] - *On medical mistrust*: “Well, sometimes…due to certain biases it doesn’t seem like all health providers have the best interest of the patients in mind or they are maybe overworked, too busy and they aren’t really taking the time to necessarily treat the patient before them. They’re treating…symptoms of…one person, or symptoms of a man that aren’t necessarily present in a woman. So it’s like when different providers are giving different information, it’s hard to, to trust it, it’s hard to know. Are you actually treating me based on…the symptoms I’m showing or are you treating me based on how you see me?” [Patient participant 12] |
|  | *Preferences for delivery of PrEP education and counseling* | - *On discussion PrEP as part of a problem-based visit (vs a follow-up visit)*: “I…like to do everything at once. I really don’t wanna…come back because I probably have… work or school or, you know, just other things. So, if it could be…a one stop shop and get everything…done. I would prefer that.” [Patient participant 14] - *On PrEP educational videos*: “I think that’s good…because [the videos] would…shift your focus off of…whatever you’re coming in for and…it’s educational. I like to…watch videos. I do think that’s better than…reading the sign.” [Patient participant 14] - *On a universal approach*: “Definitely just…erases the stigma and then makes the patient feel a lot more like, oh yeah, this is…normal to take and I’m not weird for taking this…I’m just taking care of my health and I’m doing a positive thing.” [Patient participant 4] - *On PrEP counseling*: “I think the doctor should have had a conversation as well, but maybe not up front first. Maybe the peer could be first just to give the general and the thought to start thinking of it.” [patient participant 3] - *On PrEP counseling*: “So I think the single best thing in my opinion is if the provider is like hey, like in a no judgment kind of way, you know, there’s this thing called PrEP, …you’re between these ages, I thought, you know, you might be interested in it, …what are your thoughts? I think that is the probably the single best thing…especially if there was a video before and they already kind of saw and they already kind of knew then they might be already thinking: oh, that sounds good, but ohh it might be too expensive for me or I’m scared to bring it up. So, if the provider brought it up first and they have the information, I think that would be really good.” [Patient participant 4] |
|  | *Characteristics and role of the PrEP navigator* | - *On characteristics*: “I would be more inclined if the [navigator] was woman…And I don’t know, sometimes I just feel more comfortable talking about certain things.” [Patient participant 9] - *On characteristic of being a PrEP user*: “[I] n a perfect world, I think they’d be like, yes, I use it too. But, you know, it doesn’t have to be that way for people to trust it and for people to feel like this is something I need. And this is good for me.” [Patient participant 4] - *On characteristics*: “So I would think necessarily age wouldn’t matter to me as much as like college and experience would. Someone who was very knowledgeable about…the medicine, somebody that was very knowledgeable about sexual health. They’re, the knowledge and experience for me would be a little bit more [important] because I think for particularly as it relates to age,…if…the peer educator is much older than the person receiving the information it could come off [as] preachy and not relational…I’m going with the bedside manner and knows how to kind of speak to people who is comfortable kind of engaging with a wide variety of people.” [Patient participant 12] - *On characteristics*: “[C] ommunication skills, being an open listener, so you could, you can build that trust and have that open, you know lone or flow of communication between the patient and them.” [Patient participant 11] - *On role*: “[The] educator is…giving you that introduction into what this medicine…is about, and then the…navigator is someone that’s kind of like helping you throughout the process, making sure you getting appointments and making sure you’re getting the medicine, and then the…social support is making sure that you have that extra support to make sure you feel comfortable with any issues that you have in personal or with the partnership, making sure that you’re comfortable in yourself and also with the partner that you’re with, knowing that you’re feeling grounded. So, I think all of them are more important because that introduction is that, that, that breaking that ice and being that middle navigator is making sure you have all the navigation tools to make sure that you getting’ the PrEP that you need and then that social support is making sure that you staying constant in that whole process. So, I think all of them are important to keep phoning it, but that last support is making sure that you stand steady in the process as a flow as time goes on and you’re not feeling, you know, any kind of you know, guilt or, you know, worries going further or moving forward throughout the journey so to speak.” [Patient participant 1] |

^a^KI: key informant.

^b^PrEP: pre-exposure prophylaxis.

^c^STI: sexually transmitted infection.

^d^FGD: focus group discussion.

^e^EHR: electronic health record.
